# *Cabomba caroliniana* and *Schoenoplectus californicus* as Antifouling Candidates: Anti‐Attachment and Toxicological Effects in *Aurelia coerulea* (Cnidaria, Scyphozoa)

**DOI:** 10.1002/tox.24579

**Published:** 2025-10-25

**Authors:** Mikael Luiz Pereira Morales, Laís Olivera das Neves, Ayman Shaik, Hafizah Chenia, Maximiliano Manuel Maronna, Sanye Soroldoni, Renato Mitsuo Nagata, Ng Haig They, Vanessa Ochi Agostini, Grasiela Lopes Leães Pinho

**Affiliations:** ^1^ Programa de Pós‐graduação em Oceanologia Instituto de Oceanografia (IO) da Universidade Federal do Rio Grande (FURG) Rio Grande Rio Grande do Sul Brazil; ^2^ Microbiology, School of Life Sciences University of KwaZulu‐Natal, Westville Campus Durban South Africa; ^3^ Programa de Pós‐graduação em Oceanografia Biológica Instituto de Oceanografia (IO) da Universidade Federal do Rio Grande (FURG) Rio Grande Rio Grande do Sul Brazil; ^4^ Departamento Interdisciplinar Campus Litoral Norte, Centro de Estudos Costeiros Limnológicos e Marinhos (CECLIMAR) da Universidade Federal do Rio Grande do Sul (UFRGS) Imbé Rio Grande do Sul Brazil; ^5^ Regenera Moléculas do Mar Porto Alegre Rio Grande do Sul Brazil

**Keywords:** antifouling, aquatic macrophytes, attachment, *Aurelia*, natural products, toxicology

## Abstract

Biofouling on artificial surfaces in aquatic ecosystems leads to significant economic losses. Current antifouling paints, while effective, often harm the aquatic environment. This study explores ecologically safe antifouling alternatives derived from plants, focusing on the aquatic macrophytes *Cabomba caroliniana* (CC) and *Schoenoplectus californicus* (SC). While these macrophytes have shown promise against microfouling, their effectiveness against marine macrofouling remains underexplored. For marine macrofouling tests, *Aurelia* polyps have been recommended due to their availability and handling. Using the marine cnidarian *Aurelia coerulea* (AC) as a model organism, the ability of CC and SC extracts to inhibit polyp attachment was evaluated as well as their toxicological effects on polyps and ephyrae. Additionally, the sensitivity of AC to substances (surfactant, zinc, and copper) was assessed to determine its sensitivity compared to other organisms. A dose‐dependent inhibition of polyp attachment was observed, with up to 65% efficacy. Toxicity tests indicated low toxicity at concentrations of up to 5% for CC and 20% for SC. The main compounds identified were n‐nonadecanol‐1 for CC and eicosane for SC. Additionally, AC proved to be a versatile anti‐attachment assay model, offering advantages such as sensitivity to chronic and acute tests, dual life stages, and short assay times of up to 72 h. These results suggest the biotechnological potential of CC and SC as natural antifouling agents and highlight their suitability for developing environmentally friendly antifouling applications.

## 1 Introduction

Biofouling is a complex process of ecological succession where biological deposits accumulate on submerged surfaces in aquatic ecosystems [1]. It begins with the adsorption of organic and inorganic molecules, followed by bacterial adhesion and biofilm formation [1]. Over time, this leads to colonization by other microorganisms (e.g., protozoa, fungi, and microalgae), and adhesion of macroorganisms (e.g., macroalgae, mussels, barnacles, cnidarians, and urochordates) [1, 2].

As fouling communities grow on artificial surfaces, particularly involving macrofouling [3], they alter the weight, integrity, and conformity of these structures [4]. This results in issues such as increased friction, reduced hydrodynamics, vessel buoyancy [5], and clogged water collection systems [4]. Consequently, there are large economic losses for the aquatic industries, with the estimated expense for vessel maintenance and biofouling prevention at about US$340 million [6]. Additionally, biofouling is an important vector for the spread of invasive species [3], influencing the dynamics of biological invasions.

Currently, third generation antifouling paints, containing biocides like diuron, chlorothalonil, and copper oxide [7, 8], are widely used. Limitations include being less effective at the later stages of biofouling [9] and negatively impacting aquatic ecosystems [10]. Non‐target organisms, including planktonic crustaceans and fish [5, 11], often experience toxic effects. There is thus a great need for the development of eco‐friendly “green” antifouling solutions that are safer and less harmful to the environment [12, 13], with higher biodegradability and potentially lower toxicity against non‐target organisms [12].

Aquatic macrophytes are a promising source of natural compounds with anti‐fouling potential [14, 15]. Species such as *Cabomba caroliniana* and *Schoenoplectus californicus* have shown promise due to their secondary compounds like humic acids and polyphenols [16, 17]. Morales et al. [14] reported that of 11 tested macrophyte species extracts, those from *C. caroliniana* and *S. californicus* inhibit bacterial biofilms by over 70% without harming non‐target organisms like *Thalassiosira pseudonana* and *Nitokra* species. However, their study did not evaluate the toxicity of a wide range of concentrations, only concentrations of up to 10% for *C. caroliniana* and 20% for *S. californicus* [14]. While they evaluated the anti‐microfouling effect, their efficacy against macrofouling was only studied with freshwater organisms (*Limnoperna fortunei*) [15] and was not evaluated with marine organisms.

Antifouling tests involving microfouling, particularly with bacterial biofilms, are well established [18], however, although a wide variety of organisms have been reported for macrofouling tests, most studies have been restricted to species of tubular worms and mussels [18, 19]. These species are collected in the field and exposed to chemical and physical procedures for the release of eggs and/or larvae. These procedures may take several days (~10 days) to obtain the necessary phase of their life cycle to carry out experimental tests [20]. In view of these challenges, the use of polyps of the *Aurelia* jellyfish has been gaining attention because it does not require metamorphosis to perform the anti‐attachment tests [20].

Cnidarian species of the genus *Aurelia* (Lamarck, 1816) are effective biofoulers [21], colonizing natural substrates (e.g., rocks and shells) and artificial substrates (e.g., boats and concrete structures) [22]. *Aurelia aurita* and *Aurelia coerulea* are, however, easy to cultivate with minimal laboratory conditions and are maintained in public aquariums and in laboratories around the world. These animals have been used for decades as model organisms in developmental [23], ecological [24], biomechanical [25], and ecotoxicology [26, 27, 28] studies. The availability of genetic data for multiple strains of cultures [29] along with the accumulated knowledge of physiological, reproductive, and ecological traits in various *Aurelia* species makes them excellent models for experimental biology. Furthermore, due to climate change and anthropogenic activities, in recent years the proliferation of cnidarians has been increasing, especially in estuarine regions [29].

**FIGURE 1:**
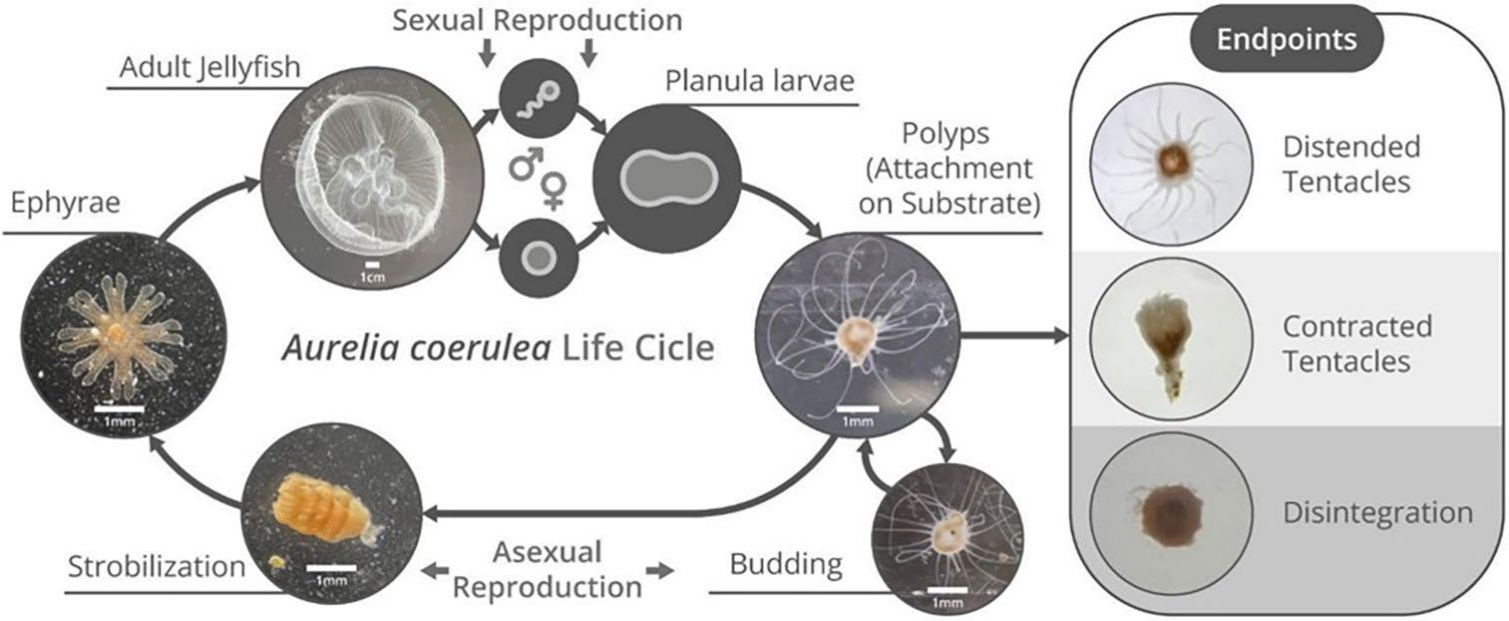
Life cycle of the scyphomedusae *Aurelia coerulea* (Cnidaria, Scyphozoa) and endpoints of toxicological tests with polyps. Credit: Hugo Custódio.

*Aurelia coerulea* , the moon jellyfish (Figure 1), offers a unique opportunity for antifouling studies due to its metogenic life cycle, with benthic (polyps) and planktonic (ephyrae and medusae) stages [30], making it ideal for testing antifouling substances. *Aurelia* ephyrae, from *A. aurita* [26, 27, 28] and *A. coerulea* [31, 32], have been widely used in toxicological studies. Studies with polyps are still incipient in relation to toxicity and anti‐attachment [20, 32]. Unlike other macrofouling organisms, *Aurelia* polyps are easy to cultivate and allow for rapid anti‐attachment testing [20]. Additionally, ephyrae and polyps exhibit varying sensitivity to stressors, enabling a comprehensive evaluation of antifouling efficacy [33].

Given the environmental concerns surrounding the use of conventional chemical antifoulants (e.g., DCOIT, Diuron, among others), the present study proposes a natural alternative antifouling based on aquatic plants, evaluating the antifouling potential and toxicological effects of *C. caroliniana* and *S. californicus* extracts on polyps and ephyres of *A. coerulea* . These plants have undergone a series of adaptations to return to the aquatic environment and ensure their survival (e.g., production of allelochemicals to defend against herbivory) [34], being strong candidates for biotechnological studies with their natural compounds. In addition, when compared to antifouling agents based on natural products of marine origin, the plants have the advantage of being easy to obtain in large quantities (collect and cultivate) [35], which helps to fill the research gap of the production of natural antifouling compounds on a large scale. In addition to this gap, we reinforce that our study provides new information about the antifouling effect of *C. caroliniana* and *S. californicus* at a more advanced stage of biofouling (macrofouling), expanding the spectrum of antifouling action of this alternative for different stages of biofouling (micro and macrofouling). In doing so, we help advance the worldwide development of environmentally sustainable antifouling approaches.

We also aimed to compare the sensitivity of *A. coerulea* to three substances – sodium dodecyl sulfate (SDS), zinc sulfate (ZS), and copper chloride (CC) – to assess their effectiveness as natural, eco‐friendly antifouling. These three substances have been used to estimate the accuracy and reliability of data produced in the laboratory [36, 37]. SDS is a surfactant generally used to assess the relative sensitivity of test organisms, as it is less toxic and easy to handle in the laboratory. Copper and zinc are commonly used substances in toxicology, as they are essential elements for organisms, but in high concentrations they are toxic [38]. These substances are widely used as base compounds for antifouling systems [39], resulting in toxicity to non‐target organisms [40]. Also, zinc and copper are widely found in antifouling paints, so using these substances ensures quality control of possible antifouling effects. We also emphasize that the substances are utilized to support comparisons across both short‐ and long‐duration tests, allow for interlaboratory standardization, and evaluate the performance of methods employing different organisms.

## 2 Methodology

### 2.1 Aurelia Species Maintenance

Polyps of *Aurelia* species were acquired through a pre‐established culture from the Zooplankton Laboratory (LABZOO) at the Universidade Federal do Rio Grande (FURG), Brazil. Polyps were maintained in plastic containers with filtered natural seawater (salinity between 32 and 35, 0.45 μm) at 20°C to 23°C under a 12L:12D photoperiod and fed *ad libitum* every 2 days with newly (< 2 days old) hatched *Artemia franciscana* nauplii. One day after feeding, the cultivation water was renewed to avoid the excessive proliferation of microorganisms. To minimize stress related to removing the polyps from the substrate, polyps were removed from the substrates 24 h before the initiation of the assays. Polyps that did not respond to stress through the contraction of their tentacles and did not attach to the substrate were selected for the assays.

To obtain ephyrae from *Aurelia* polyps, strobilation was induced by acclimation in decreased temperature to ~15°C in 1.5 L plastic containers with artificial 35 salinity water (ASW) and a photoperiod of 12 L:12D (adapted from [26]). Once the ephyrae (0 to 5 days old) were released, they were immediately collected and placed in glass beakers to carry out toxicological tests. The ephyrae were fed *ad libitum* with *A. franciscana* nauplii, 24 h before the tests, to increase swimming activity.

### 2.2 Molecular Species Determination

Total DNA was extracted from three whole‐body polyps cultivated at LABZOO at FURG with an ammonium acetate protocol (triplicates [41]). For taxonomic validation of analyzed cultures, selected molecular markers were amplified and sequenced: from the mitochondrial genome, a ~650 bp fragment of the large ribosomal 16S rRNA subunit and ~650 bp of the protein‐coding COI I subunit [42]; from their nuclear genome, a ~650 bp fragment of the large ribosomal 28S rRNA subunit [43]. Amplification based on Polymerase Chain Reaction (PCR) protocols followed standard procedure, and thermocycler reaction conditions were conducted as described by Lawley et al. [30]. PCR products were purified using the Agencourt AMPure XP kit (B37419AB), and the BigDye reactions used the same primers and Tm conditions as original PCRs. Finally, these amplicons were precipitated (sodium acetate and ethanol) and sequenced using an ABI PRISM 3100 Hitachi genetic analyzer. Using Geneious 9.5 [44], chromatograms were assembled, trimmed, aligned, and final consensus sequences were compared with data available in GenBank to identify the *Aurelia* species [30]. Sequence data was deposited in the NCBI database.

### 2.3 Test Solutions

To verify the sensitivity of the two *Aurelia* life stages (polyp and ephyrae), toxicology assays were carried out with the substances the surfactant sodium dodecyl sulfate (SDS—NaC_12_H_25_SO_4_), zinc sulfate (ZS—ZnSO_4_), and copper chloride II (CC—CuCl_2_) (Labsynth). For this, SDS in its powder form was used to prepare diluted treatments in ASW with salinity 35 (Marine salt VeroSal Corais). The concentrations were 5, 15, 45, and 135 mg·L^−1^ of SDS for polyps and 0.5, 1, 2.5, 5, and 15 mg··L^−1^ for ephyrae. Powdered ZS and CC were also used to prepare treatments with ASW. The defined concentrations were as follows: ZS—1, 2, 3, 4, and 5 mg·L^−1^ for polyps and 0.4, 0.8, 1.6, 2.4, and 3.2 mg·L^−1^ for ephyrae and CC—0.1, 0.25, 0.5, 1, and 2.5 mg···L^−1^ for polyps and 0.02, 0.05, 0.10, 0.15, and 0.20 mg·L^−1^ for ephyrae. These concentrations were defined based on the literature with tests with different organisms [27, 45, 46], as well as having different concentration gradients to calculate LC_50_ (lethal concentration for 50% of the population) and EC_50_ (effect concentration for 50% of the population).

Aqueous extracts of *C. caroliniana* (stalk and leaf) and *S. californicus* (inflorescence and stalk) were prepared according to the methodology described by Morales et al. [14]. Plant biomass was collected in permanent lakes in southern Brazil (32° 09′ 23.3″ S 52° 05′ 57.6″ W), dried oven at 60°C–80°C until it reached a constant mass, and manually crushed with a pestle and mortar. To prepare the extracts, 6 g of dry biomass were mixed with 300 mL of ASW (salinity 35) and left to rest for 24 h at 22°C. Thereafter, the mixture (total) was centrifuged and sterilized by filtration (0.2 μm) (cellulose acetate filter, Sartorius Biolab Products). Preparation of the extracts resulted in a 100% stock solution, which was used to obtain test concentrations through dilution in sterile ASW. The concentrations defined for the tests were 5%, 10%, 20%, 40%, and 80%. Treatments with SDS, ZS, CC, and aquatic macrophyte extracts were used to carry out attachment and toxicological assays under experimental conditions of 20°C, 12 L:12D. For both experiments, controls were set by ASW with salinity 35.

### 2.4 Toxicological Assays

#### 2.4.1 Assays With Polyps

Polyps were individually isolated in 6‐well plates, each well containing 10 mL of treatment. For each treatment, three replicates were prepared, each containing four polyps. After 1, 6, 24, 48, 72, and 96 h of exposure, chronic (sublethal) and acute responses were observed with the aid of a stereoscopic microscope Olympus SZX9, magnification 40× (Table 1).

**TABLE 1:** *Aurelia coerulea* selected endpoints.

Life stage	Assays		Observation time (h)
	Acute	Chronic (sublethal)	
Polyps	Disintegration	Contraction of the tentacles	1, 6, 24, 48, 72 and 96
		Capture and ingestion	48 and 96
Ephyrae	Immobility	Pulsation frequency	24 and 48

The following chronic endpoints were observed: tentacle contraction by *Aurelia* species was indicated as distended (all tentacles were distended, even if slightly curved; Figure 1) or contracted tentacles (when all tentacles were absolutely contracted; Figure 1); change in prey ingestion was observed during 30 s of food capture and ingestion (% of individuals who ingest prey). Feeding *ad libitum* was done by adding 1 mL of solution containing *A. franciscana* nauplii in a controlled and progressive manner, using a Pasteur pipette. Observation of the change in diet was carried out at 48 and 96 h. The acute response was indicated by mortality determined by the observation of total or partial disintegration of the polyps (Figure 1). A polyp is considered to disintegrate when it loses its typical body shape, which results in tissue fragmentation (Figure 1).

#### 2.4.2 Assays With Ephyrae

Ephyrae were exposed to each treatment in 24‐well plates, each well containing 2 mL of the treatment (one individual per well). For each treatment, three replicates were prepared, each containing four individuals, one per well to avoid interactions between organisms [27]. After 24 and 48 h, acute and chronic (sublethal) effects were observed (Table 1).

The acute response for each treatment was observed through the mobility of the organisms, by observation of the individual's movement for 10 s after stimulation with the aid of a Pasteur pipette under a stereoscopic microscope (Olympus SZX9, magnification 40×). Completely immobile ephyra (unable to change their barycenter position during 10 s) were counted as immobile organisms, and the percentage of immobility (%) was calculated for each treatment compared to the control treatment. The chronic response was given by the pulsation frequency (PF) of each ephyra, measured by observing it under a stereoscopic microscope. For each ephyra, three PF measurements of 10 s each were performed. The average of the three measurements was calculated and the data transformed to PF per minute for each treatment.

### 2.5 Attachment Assays

Polyps were individually exposed to the three substances and two extract treatments in 6‐well plates containing 10 mL of treatment and one individual per well. For each treatment, three replicates were prepared, each assessing four polyps. Polyp attachment was evaluated over periods of 24, 48, 72, and 96 h by the polyps' resistance to detaching from the substrate when subjected to light water flows performed with the aid of a Pasteur pipette. In addition, we confirmed the attachment of the organisms with the aid of a stereoscope microscope (Olympus SZX9, magnification 40×). Each individual's attachment was counted for all treatments and control, and the results were expressed as a percentage of attachment (%). The polyps accounted for in this stage were only those that were not disintegrated, that is, those that were alive and had the ability to adhere or not.

### 2.6 Chemical Characterization of Extracts

#### 2.6.1 Gas Chromatography–Mass Spectroscopy (GC–MS)

The two‐crude aqueous macrophyte extracts were subjected to gas chromatography–mass spectroscopy (GC–MS) to identify their composition. A 1 μL solution was injected into a Shimadzu gas chromatograph (series AOC‐20i)–coupled mass spectrophotometer (GCMS‐QP2010 SE). Helium was used as the carrier gas. The column oven temperature was programmed at 50°C and the injection temperature was 260°C, while the flow rate was 0.68 mL/min. The capillary column used was Zebron ZB‐5MSplus column 0.25 × 30 m (length) × 0.25 μm (df). A 50/50 split ratio was used for the injection sample at an initial holding time of 1 min, then later at 10 min. The start time for analysis was 3 min, while the end time was 32 min. The spectra were set at 20 to 1000 m/z to avoid capturing water molecules and other volatiles by the GC. Analysis commenced after 3 min and ended after 32 min, with the spectra set at 20–1000 m/z to prevent interference by water molecules and other volatiles from the gas chromatograph. The relative amount of each phyto‐component present in extracts was expressed as a percentage based on peak height (%) produced in the chromatograph. We emphasize that this evaluation is initial, and that each extract was analyzed only once, with no estimate of standard error of the molecules found, as well as their actual quantification in concentration. The recorded mass spectra of the constituents of the crude extracts were identified using the standard mass spectra from the National Institute of Standards and Technology (NIST05.LIB) library data provided by the GC–MS system software [47, 48].

#### 2.6.2 Liquid Chromatography‐Mass Spectroscopy (LC–MS)

Liquid chromatography‐mass spectrometry (LC–MS) data was obtained using a Shimadzu LCMS2020 with a Shimadzu Shim‐Pak GIST HP C18 3 μm 4.5 × 150 mm column equipped with a UV detector using a mobile phase of 20%–90% acetonitrile, 10% water, and both containing 1.1% formic acid at a flow rate of 1 mL/min. The recorded mass spectra of the constituents of the crude extracts were identified using the standard mass spectra from the analytical data MASSBANK (http://www.massbank.jp/index‐e.html) [49]. We highlight that this represents an initial screening, with each extract subjected to a single analysis, lacking both error estimates and precise quantification of the concentration of the detected compounds.

#### 2.6.3 Fourier Transmission Infrared Spectroscopy (FTIR)

Fourier transmission infrared spectroscopy (FTIR) was performed to identify the functional groups present in the two crude extracts. Crude extract samples were prepared for analysis by drying 2 mg of the extracts, which were mixed with 200 mg KBr. Crude extracts were analyzed using the Bruker Alpha II Infrared Spectrophotometer (Parameters: ATR Diamond^−1^ Bounce, 24 background scans, 24 sampling scans, 4000–400 cm^−1^ range with a resolution of 4 cm^−1^) and processed with Opus Spectroscopy Software. Functional groups of the crude biosurfactants were identified using IR spectrum tables provided by Sigma‐Aldrich [47, 48].

### 2.7 Statistical Analysis

For the purposes of experimental reproducibility, each experiment (exposure to the extracts and substances) was performed twice. Each of the experiments had 3 replicates per treatment, with each replication involving 4 subjects, totaling 12 subjects per treatment. This reproducibility standard was used for all assays with polyps and ephyrae; the results were presented as means and standard deviations. For acute responses, LC_50_ values were calculated over a period of 96 h for polyps and EC_50_ values of 48 h for ephyrae, using Probit Analysis [50]. To test possible differences between treatments at each exposure time for polyps and mobility of ephyrae, generalized linear models (GLM) were used. Because these variables are discrete, a model with a binomial distribution was used. As for the ephyrae pulsation frequency, as this variable is continuous, the normality and homoscedasticity of the residuals were verified using the Shapiro–Wilk and Levene tests, respectively. With the acceptance of the premises, a one‐way ANOVA was used. When a significant effect of a variable was detected, a *post hoc* Tukey comparison was performed to detect significant differences between levels of the factors. Statistical analyses were performed using GraphPad Prism 8.4 software for Windows (San Diego, USA).

## 3 Results

### 3.1 Molecular Identification

Genetic sequences confirmed that the *Aurelia* culture used belongs to *A. coerulea* von Lendenfeld, 1884 (+98% DNA sequence homology identity for all three molecular markers with samples from the Mediterranean Sea).

### 3.2 Substances Sensitivity

#### 3.2.1 Attachment Assays

**FIGURE 2:**
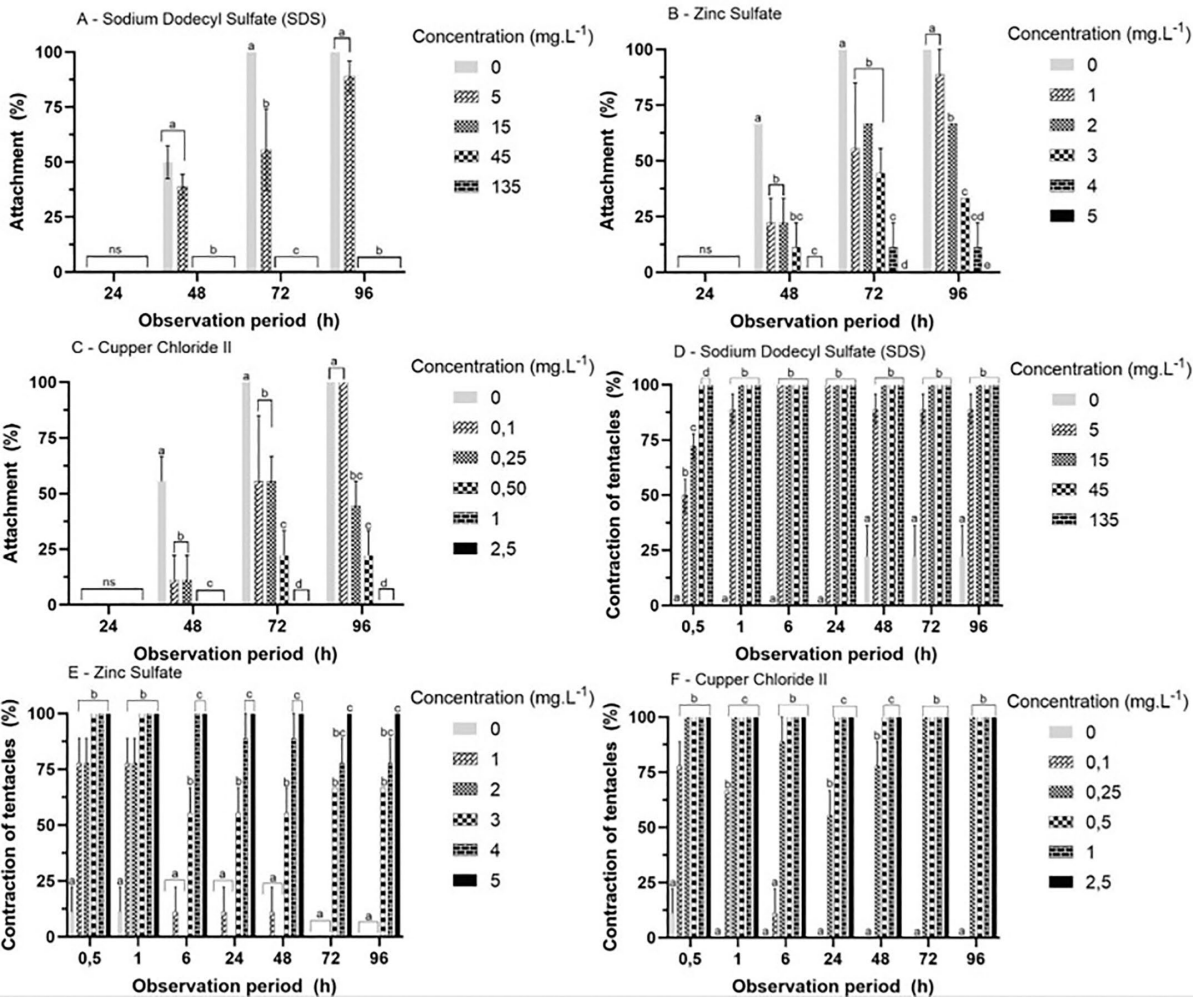
Mean (± SD) of attachment (A–C) or tentacle contraction (D–F) of *Aurelia coerulea* polyps exposed to reference substance at different times. Different letters within each observation period: Statistical difference between concentrations (*p* < 0.05), visualized by generalized linear model (GLM) and Tukey post hoc multiple comparison. ns = There was no significant difference between treatments.

For all test solutions, polyp attachment began after 48 h, stabilizing after 72 h (Figure 2A–C and Table S1). Attachment was inhibited from 15 mg·L^−1^ (*p* < 0.05) when testing the surfactant SDS (Figure 2A). With ZS and CC (Figure 2B,C), exposure to 1 and 0.1 mg·L^−1^, respectively, did not inhibit attachment (*p* > 0.05); however, the remaining higher concentrations inhibited attachment in a dose‐dependent manner (*p* < 0.05).

#### 3.2.2 Toxicological Assays With Polyps

After 1 h, polyps achieved 100% tentacle contraction at all SDS concentrations (Figure 2D) except the control (*p* < 0.05). This response was observed at all subsequent exposure times. For ZS (Figure 2E), tentacle contraction occurred in a dose‐dependent manner after 6 h of exposure (*p* < 0.05). For CC, the tentacle contraction response pattern was the same as observed for SDS (Figure 2F). From 72 h onwards, all ZS concentrations (Figure 2E) showed 100% contraction (*p* < 0.05).

**FIGURE 3:**
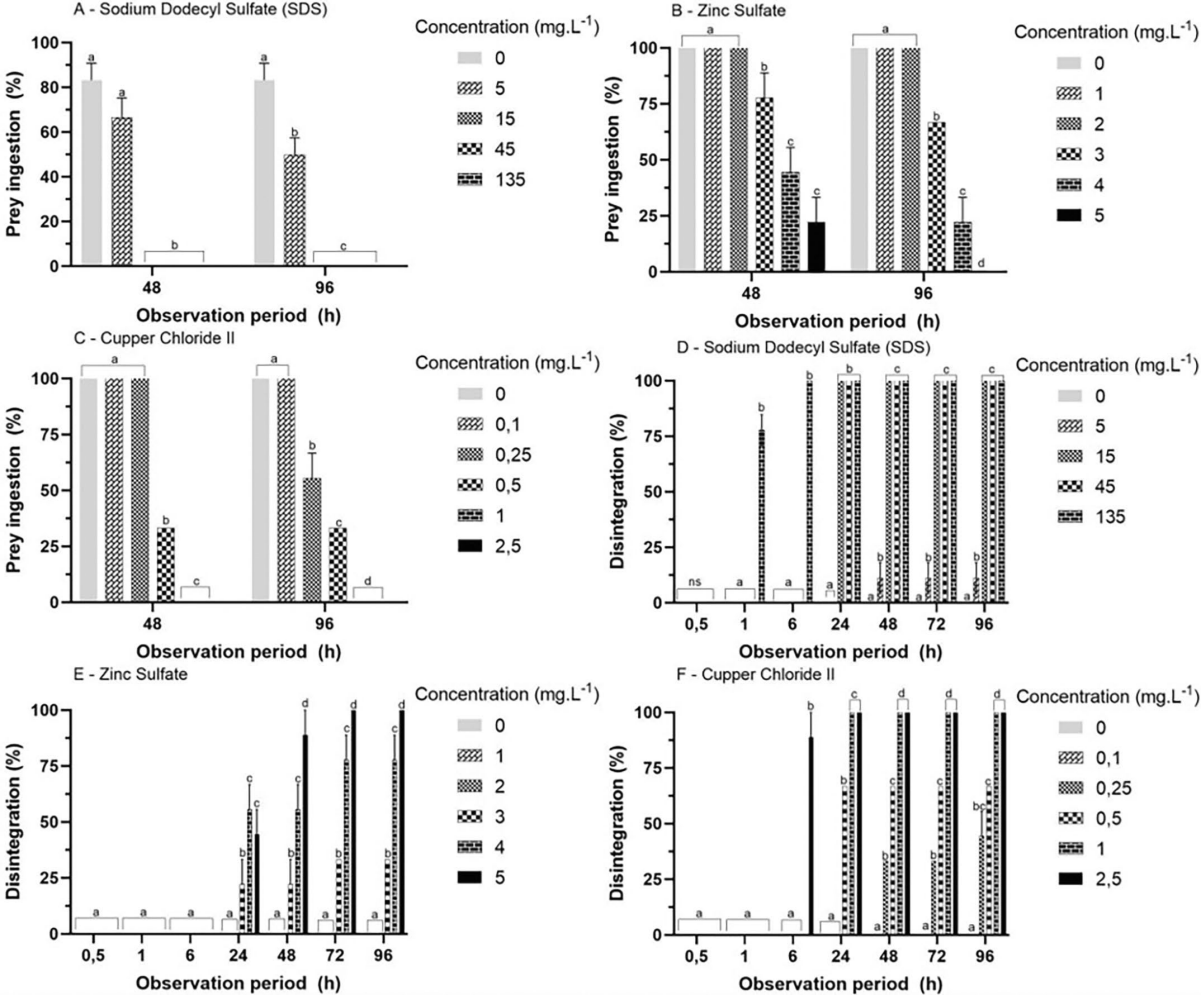
Mean (± SD) of the response prey ingestion (A–C) and dead (disintegration; D–F) of polyps of *Aurelia coerulea* following exposure to reference substance. Different letters within each observation period: Statistical difference between concentrations (*p* < 0.05), visualized by generalized linear model (GLM) and Tukey post hoc multiple comparison.

The responses of the prey ingestion assay were similar between 48 and 96 h (Figure 3) for all test solutions. For SDS (Figure 3A), the percentage of prey ingested decreased from ≥ 5 mg·L^−1^ (*p* < 0.05). For ZS and CC, prey ingestion decreased with increasing concentration (*p* < 0.05), demonstrating a dose‐dependent response (Figure 3B,C).

For all test solutions, the disintegration response increased after 48 h of exposure, remaining constant at 72 and 96 h (Figure 3). For SDS at ≥ 15 mg·L^−1^, disintegration was 100% after 24 h (Figure 3D). For ZS and CC (Figure 3E,F), the disintegration response also occurred in a dose‐dependent manner after 24 h (*p* < 0.05). The LC_50_ for SDS, ZS, and CC was 8.66, 3.33, and 0.30 mg·L^−1^, respectively (Table 2).

**TABLE 2:** LC_50_ or EC_50_ values for different marine test organisms following exposure to sodium dodecyl sulfate (SDS), zinc sulfate, copper chloride II and aqueous aquatic macrophyte extracts ( *Cabomba caroliniana* and *Schoenoplectus californicus* ).

Marine organisms	Species	Habitat	Substance	LC_50_ or EC_50_ (mg·L^−1^)	Confidence interval (95%)		Exposure time (h)	Authors
					Minimum	Maximum		
Crustacea	*Amphibalanus amphitrite* (larvae)	Planktonic	SDS	7.49	No information	No information	48	Greco et al. [51]
Mollusca	*Perna perna* (larvae)	Planktonic	SDS	0.86	0.79	0.91	48	Jorge and Moreira [37]
Echinodermata	*Paracentrotus lividus* (sperm)	Planktonic	SDS	3.18	3.18	3.89	24	Mariani et al. [52]
Crustacea	*Artemia salina* (nauplius)	Planktonic	SDS	34.15	28.7	40.64	24	Rotini et al. [53]
Crustacea	*Tigriopus fulvus* (nauplius)	Benthic	SDS	8.52	8.00	9.17	96	Mariani et al. [52]
Fish	*Dicentrarchus labrax* (juveniles)	Planktonic	SDS	7.88	7.10	8.19	48	Mariani et al. [52]
Cnidaria	*Aurelia* sp. (ephyra)	Planktonic	SDS	1.55	No information	No information	48	Faimali et al. [27]
Cnidaria	*Aurelia coerulea* (ephyra)	Planktonic	SDS	1.00	0.28	2.56	48	**This study**
Crustacea	*Monokaliapseudes schubarti* (adult)	Planktonic	SDS	56.57	46.55	68.74	96	Perina et al. [11]
Crustacea	*Acartia tonsa* (adult)	Planktonic	SDS	2.08	1.84	3.34	48	Perina et al. [11]
Crustacea	*Tiburonella viscana* (adult)	Benthic	SDS	12.84	9.93	16.60	96	Perina et al. [11]
Cnidaria	*Aurelia coerulea* (polyp)	Benthic	SDS	8.66	6.24	12.00	96	**This study**
Mollusca	*Perna perna* (larvae)	Planktonic	Zinc Sulfate	0.54	0.50	0.57	48	Jorge and Moreira [37]
Crustacea	*Parhyale hawaiensis* (neonates)	Planktonic	Zinc Sulfate	1.73	1.42	2.09	96	Artal et al. [54]
Crustacea	*Artemia salina* (nauplius)	Planktonic	Zinc Sulfate	10.00	5.00	13.00	48	Dobretsov et al. [55]
Cnidaria	*Aurelia coerulea* (ephyra)	Planktonic	Zinc Sulfate	2.72	2.24	3.31	48	**This study**
Crustacea	*Nitokra* sp.(adult)	Benthic	Zinc Sulfate	0.69	0.60	0.78	168	Artal et al. [54]
Cnidaria	*Aurelia* sp. (polyp)	Benthic	Zinc Sulfate	3.33	2.98	3.72	96	**This study**
Crustacea	*Eurytemora affinis* (nauplius)	Planktonic	Copper Chloride II	0.15	0.01	0.34	96	Heuschele et al. [40]
Crustacea	*Tigriopus fulvus* (nauplius)	Benthic	Copper Chloride II	0.05	0.01	0.01	96	Rinna et al. [56]
Cnidaria	*Aurelia coerulea* (ephyra)	Planktonic	Copper Chloride II	0.023	0.016	0.033	48	**This study**
Crustacea	*Acartia tonsa* (adult)	Planktonic	Copper Chloride II	0.12	0.05	0.20	48	Pinho et al. [45]
Crustacea	*Nitokra spinipes* (adult)	Benthic	Copper Chloride II	1.95	1.74	2.16	96	Heuschele et al. [40]
Crustacea	*Eurytemora affinis* (adult)	Benthic	Copper Chloride II	0.09	0.01	0.18	96	Heuschele et al. [40]
Cnidaria	*Aurelia coerulea* (polyp)	Benthic	Copper Chloride II	0.30	0.18	0.48	96	**This study**
Cnidaria	*Aurelia coerulea* (ephyra)	Planktonic	*C. caroliniana*	62.92	51.45	79.07	48	**This study**
Cnidaria	*Aurelia coerulea* (ephyra)	Planktonic	*S. californicus*	10.14	6.14	16.74	48	**This study**
Cnidaria	*Aurelia coerulea* (polyp)	Benthic	*C. caroliniana*	87.49	74.68	101.51	96	**This study**
Cnidaria	*Aurelia coerulea* (polyp)	Benthic	*S. californicus*	10.76	6.18	18.72	96	**This study**

#### 3.2.3 Toxicological Assays With Ephyrae

**FIGURE 4:**
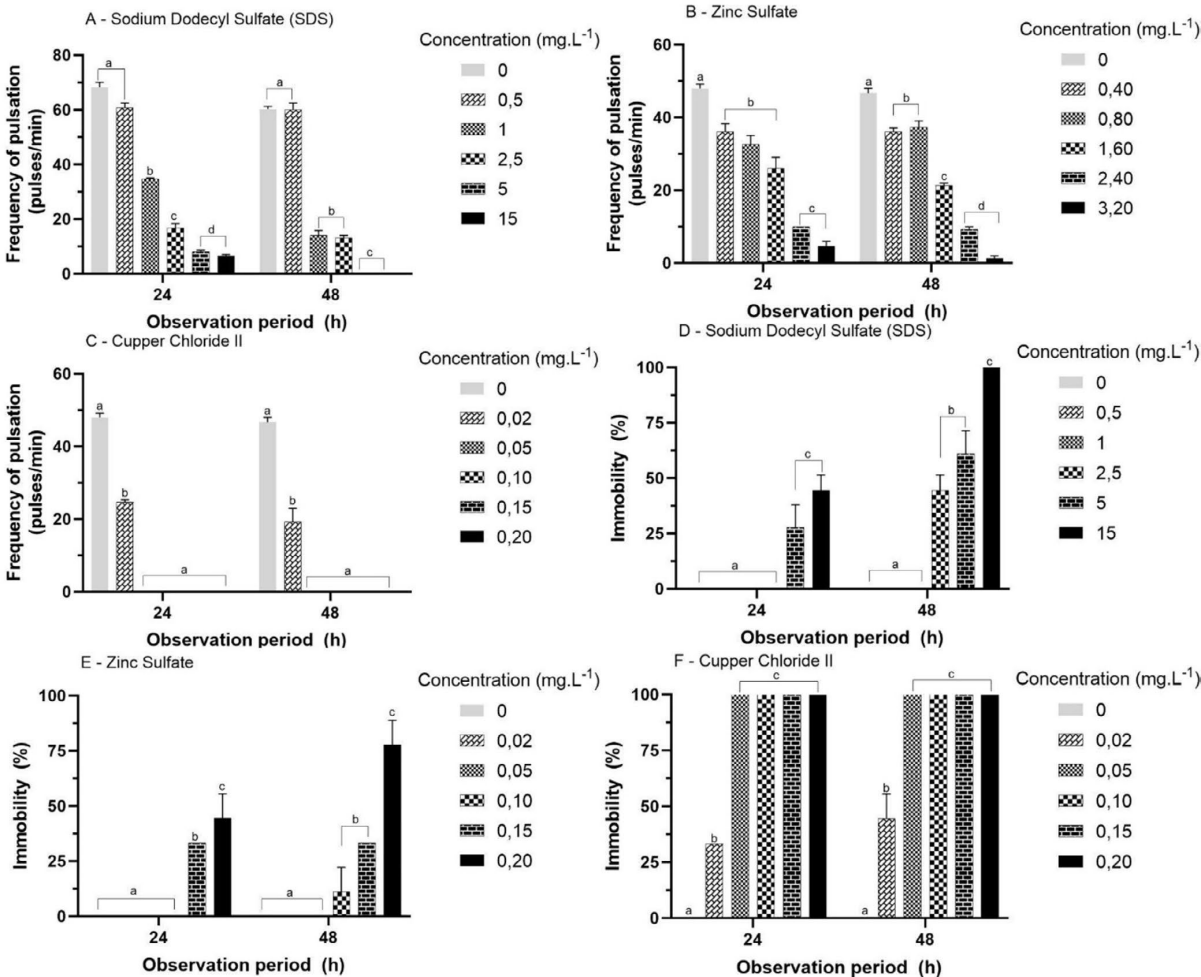
Mean (± SD) of the frequency of pulsation (A–C) and immobility (D–F) response of ephyrae of *Aurelia coerulea* exposed to different reference substances. Different letters within each observation period: Different between concentrations (*p* < 0.05), visualized by one‐way ANOVA and Tukey post hoc multiple comparison.

Pulsation frequency responses were similar for 24 and 48 h exposure to all solutions, presenting a dose‐dependent response (Figure 4). For SDS (Figure 4A), the PF decreased from the concentration of 1 mg·L^−1^ (*p* < 0.05). For ZS (Figure 4B), the frequency of pulsation decreased from the concentration of 0.4 mg·L^−1^ and for CC (Figure 4C) to a concentration of 0.02 mg·L^−1^.

Immobility responses were similar for 24 and 48 h across all tested solutions (Figure 4). For SDS and ZS (Figure 4D,E), the immobility response was dose‐dependent (*p* < 0.05). For CC (Figure 4F), immobility was significantly higher than the control for all concentrations tested (0.02 mg/L). The EC_50_ values were 1.00, 2.72, and 0.023 mg·L^−1^ for the SDS, ZS, and CC, respectively (Table 2).

### 3.3 Extracts of Aquatic Macrophytes

#### 3.3.1 Attachment Assays

**FIGURE 5:**
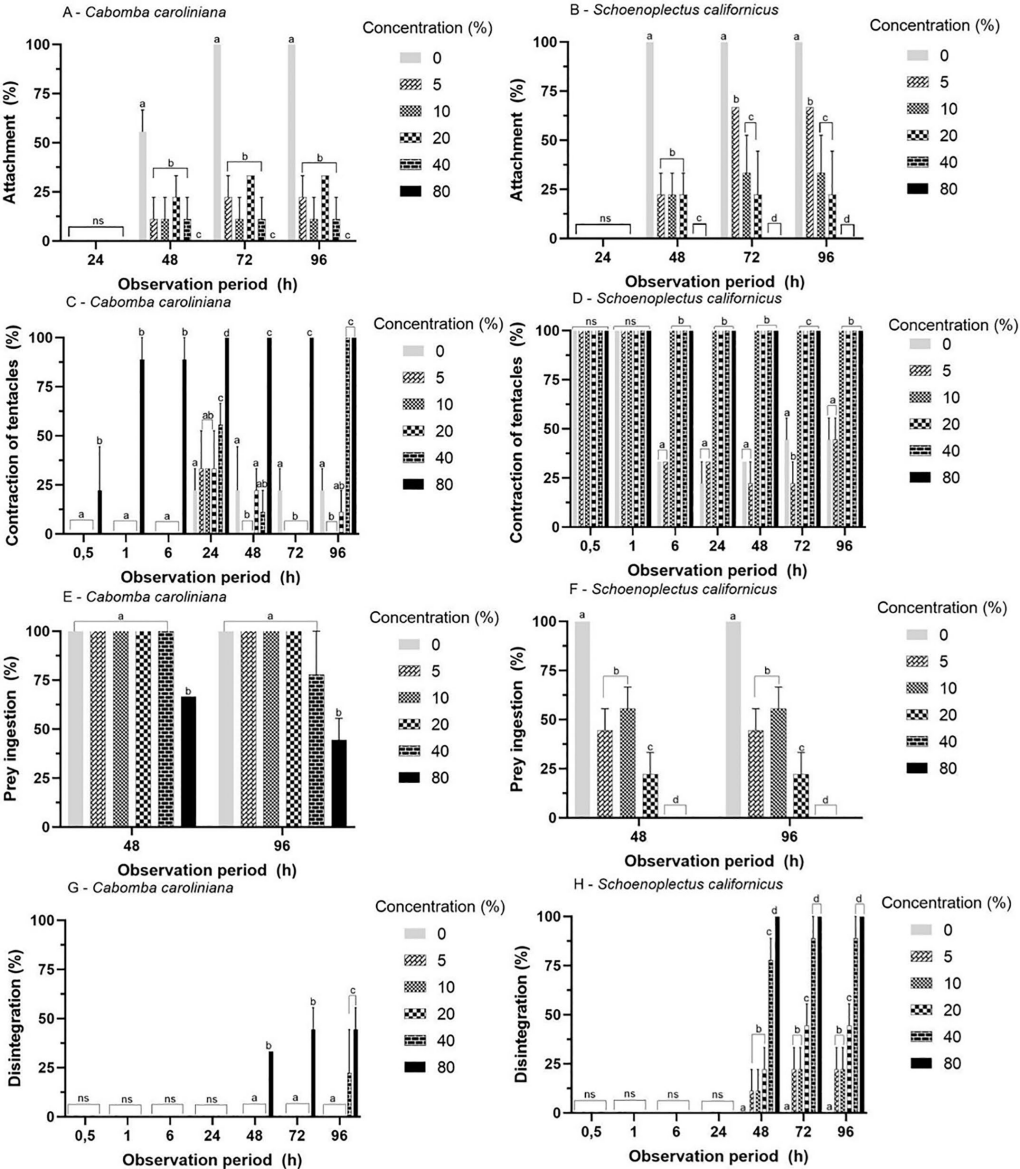
Mean (± SD) of the attachment (A, B) contraction of tentacles (C, D), prey ingestion (E, F), and dead (disintegration; G, H) response of polyps of *Aurelia coerulea* exposed to aqueous extracts of aquatic macrophytes *Cabomba caroliniana* and *Schoenoplectus californicus* . Different letters within each observation period: Statistical difference between concentrations (*p* < 0.05), visualized by generalized linear model (GLM) and Tukey post hoc multiple comparison.

For the *C. caroliniana* extract, the 80% concentration inhibited attachment at all exposure times (Figure 5A and Table S1; *p* < 0.05), while the other concentrations showed attachment inhibition ≥ 65% after 48 h (*p* < 0.05). The attachment of polyps exposed to *S. californicus* demonstrated a dose‐dependent response (*p* < 0.05), decreasing under higher concentrations (Figure 5B).

#### 3.3.2 Toxicological Assays With Polyps

For *C. caroliniana* , there was an increase in tentacle contraction with increasing extract concentrations (*p* < 0.05) at 24 and 48 h (Figure 5C). However, at 96 h (Figure 5C), there was a decrease in tentacle contraction (≤ 25%) at concentrations of 5% and 10%, compared to the control (*p* < 0.05). For the *S. californicus* extract (Figure 5D), the response remained constant after 6 h of exposure, with 100% contraction at concentrations of 10%, 20%, 40%, and 80% (*p* < 0.05). For the *C. caroliniana* extract (Figure 5E), prey ingestion decreased by 50% only at the 80% concentration (*p* < 0.05). For the *S. californicus* extract, prey ingestion decreased with increasing concentrations (*p* < 0.05), demonstrating a dose‐dependent response (Figure 5F).

For the *C. caroliniana* extract (Figure 5G), disintegration occurred only at 40% and 80% concentrations (*p* < 0.05). As for the *S. californicus* extract, disintegration increased with increasing extract concentration after 48 h of exposure (*p* < 0.05), reaching 100% disintegration at concentrations of 40% and 80% (Figure 5H). This response remained constant for the 72 and 96 h periods (Figure 5G,H). The LC_50_ for the *C. caroliniana* extract was 62.92%, while that of *S. californicus* was 10.76% (Table 2).

#### 3.3.3 Toxicological Assays With Ephyrae

**FIGURE 6:**
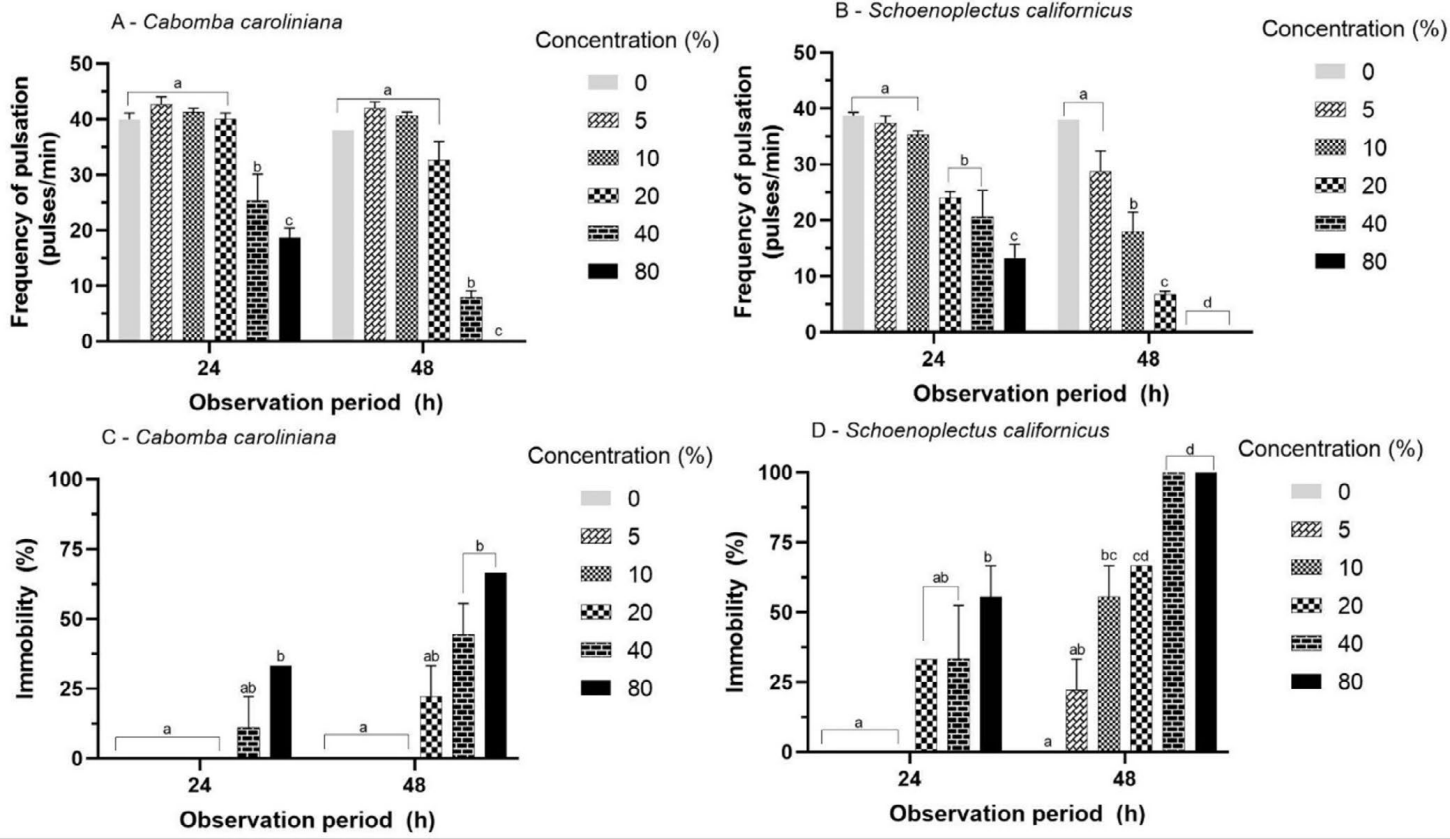
Mean (± SD) of the frequency of pulsation (A, B) and immobility (C, D) response of ephyrae of *Aurelia coerulea* exposed to different extracts of aquatic macrophytes *Cabomba caroliniana* and *Schoenoplectus californicus* . Different letters within each observation period: Statistical difference between concentrations (*p* < 0.05), visualized by one‐way ANOVA and Tukey post hoc multiple comparison.

For the *C. caroliniana* extract (Figure 6A), concentrations of 5%, 10%, and 20% had no effect on the PF compared to the control (*p* > 0.05). For the *S. californicus* extract (Figure 6B), a dose‐dependent response was observed with the PF decreasing with increasing concentrations (*p* < 0.05).

Immobility responses were similar for 24 and 48 h exposures to all test solutions (Figure 6C,D). For *C. caroliniana* and *S. californicus* extracts (Figure 6C,D), the immobility response was dose‐dependent (*p* < 0.05). The EC_50_ for the *C. caroliniana* extract was 62.92%, while that of the *S. californicus* extract was 10.14% (Table 2).

### 3.4 Chemical Characterization of Extracts

#### 3.4.1 Gas Chromatography–Mass Spectroscopy (GC–MS)

The three most abundant compounds identified in the *C. caroliniana* extract were n‐nonadecanol‐1 with 24.39% area, cyclohexadecane with 19.42%, and eicosane with 12.78% (Table 3A). The three most abundant compounds in the *S. californicus* extract were eicosane with 32.90%, E‐15‐heptadecenal with 16.08%, and 9‐tricosene (Z) with 13.94% (Table 3A). Complete data such as retention time and chromatographic peaks are presented in the Supporting Information (Table S1 and Figure S1).

**TABLE 3:** GC–MS (A) and LC–MS (B) analysis of aqueous extract of aquatic macrophytes ( *Cabomba caroliniana* and *Schoenoplectus californicus* ).

(A) GC–MS analysis				
Compound	Molecular formula	*C. caroliniana* extract	*S. californicus* extract	Chemical group
		Area %	Area %	
n‐Nonadecanol‐1	C_19_H_40_O	24.39	3.42	Alcohol
Cyclohexadecane	C_16_H_32_	19.42	—	Ciclic Alkane
Eicosane	C_20_H_42_	12.78	32.90	Alkane
E‐15‐heptadecenal	C_17_H_32_O	—	16.08	Aldehyde
9‐tricosene	C_23_H_46_	—	13.94	Alkene

(B) LC–MS analysis				
Compound	Molecular formula	*C. caroliniana* extract	*S. californicus* extract	Chemical group
		Area %	Area %	
4‐Methylphenethylamine	C_9_H_13_N	77.85	73.83	Aromatic compound
Diethanolamine	C_4_H_11_NO_2_	19.35	—	Amines
Lysine hydrochloride	C_6_H_14_N_2_O_2_ClH	1.04	0.03	Amino acid
L‐Lysine monohydrochloride	C_6_H_15_ClN_2_O_2_	—	13.79	Amino acid
Piperidinic acid	C_4_H_9_NO_2_	—	6.36	Carboxylic acid

#### 3.4.2 Liquid Chromatography‐Mass Spectroscopy (LC–MS)

The three compounds abundant in the *C. caroliniana* extract were 4‐methylphenethylamine with mean 77.85% area, diethanolamine with mean 19.35%, and lysine hydrochloride with mean 1.04% (Table 3B). The abundant compounds in the *S. californicus* extract were 4‐methylphenethylamine with mean 73.83%, L‐lysine monohydrochloride with mean 13.79%, and piperidinic acid with mean 6.36% (Table 3B). Complete data such as retention time and chromatographic peaks are presented in the Supporting Information (Tables S2, S2, and Figure S2).

#### 3.4.3 Fourier Transmission Infrared Spectroscopy (FTIR)

FTIR indicated different functional groups for the two extracts (Table S3). For the *C. caroliniana* extract, the functional groups identified included primary amine, aldehyde, amide, and fluor compounds. The *S. californicus* extract mainly presented functional groups such as amide, alcohol‐hydrogen bonded, alkyne, alcohol, aliphatic ether, and halo compound.

## 4 Discussion

Overall, the results of this study underscore the potential of aquatic macrophyte‐derived extracts as effective and environmentally friendly antifouling agents. Interest in plant‐derived antifouling alternatives has grown significantly, with a particular focus on phytochemicals as potential antifouling agents [18]. However, research on aquatic macrophyte extracts for biofouling control remains in its early stages, particularly regarding their effects on macrofouling organisms [14, 15]. In the present study, the antifouling efficacy of aqueous extracts of *C. caroliniana* and *S. californicus* was evaluated by examining their ability to inhibit the attachment of *A. coerulea* polyps, as well as their toxicity to polyps and ephyrae.

Recent studies have shown promising antifouling effects of macrophyte extracts. For instance, these *C. caroliniana* and *S. californicus* extracts have demonstrated up to 70% biofilm inhibition for single‐ and multi‐species estuarine bacteria [14]. For this study, a dose‐dependent anti‐attachment response was observed for *C. caroliniana* and *S. californicus* extracts, aligning with other studies investigating plant‐derived anti‐macrofouling agents. Pinteus et al. [20] observed that extracts of the algae *Asparagopsis armata* and *Sargassum muticum* showed an inhibitory effect on the attachment of *A. aurita* polyps. Feng et al. [57] observed that 15 alkaloids extracted from plants inhibited the attachment of *Bugula neritina* and *Fistulobalanus albicostatus* larvae. Similarly, *Verbena bonariensis* and *Tillandsia tenuifolia* extracts effectively deterred macrofouling of adult *Mytilus eduli*s [12]. Moreover, *Halophila stipulacea* extracts have shown antifouling activity against bacteria and *Mytilus galloprovincialis* , with a dose‐dependent effect [58]. Additionally, *Posidonia oceanica* extracts reduced diatom and bacterial biofilms while also inhibiting polychaete *Ficopomatus enigmaticus* adhesion [59].

Unlike barnacles, mussels, and polychaetes, the use of polyps in antifouling research is relatively novel. To date, only studies by Pinteus et al. [20] and the present work have focused on this application. By analyzing the DNA sequence, it was possible to verify the identity of the study polyps as *A. coerulea* . This species is known for its wide geographic distribution and numerous introductions, as detailed by Lawley et al. [30]. For assays with *A. coerulea* , it is not necessary to perform a metamorphosis step since laboratory cultures are usually kept in the polyp phase. Thus, obtaining data through macrofouling tests with this species can be much faster (3 days of testing) compared to tests performed with other macrofouling species. Moreover, *A. coerulea* polyps multiply readily via asexual reproduction, allowing for a rapid increase in available specimens under adequate feeding and space conditions [60].

In our study, *A. coerulea* polyp attachment stabilized within 72 h, making assays with this species significantly faster than with others, such as *A. aurita* or *Phyllorhiza punctata* , which require up to 120 and 144 h, respectively [20]. This efficiency, coupled with the straightforward cultivation of *A. coerulea* polyps, underscores their practical advantages for antifouling research. In contrast, the utilization of mussel and barnacle larvae involves time‐consuming and often unsuccessful procedures to release and develop larvae in the pre‐nesting stage, causing a delay of at least a few days (e.g., 6 days for barnacles) to start experiments [61]. For barnacles, after obtaining the larvae, antifouling tests usually last three days [48], often requiring up to 9 days to complete the trial.

We emphasize that in addition to having endpoints to be used in *Aurelia* anti‐attachment tests, due to its metagenic life cycle, it is also possible to use them in toxicological tests with different life stages (polyps and ephyrae), which guarantees its scope in the performance of tests for the development of new antifouling agents. Toxicological tests during the settlement stage are crucial to confirm that attachment inhibition results from attachment inhibition rather than extracting toxicity. In this study, the *S. californicus* extract demonstrated significantly higher toxicity than the *C. caroliniana* extract at both life stages. The non‐toxic concentration for the *S. californicus* extract was 5%, compared to 20% for the *C. caroliniana* . This was evident in chronic behavioral responses such as feeding and tentacle contraction in polyps, PF responses in ephyrae, and acute lethality responses. These results align with previous findings on these extracts' effects when evaluating cell density and chlorophyll‐a content of *T. pseudonana* and *Nitokra* species survival [14]. In the present study, at non‐toxic concentrations, the extracts inhibited *A. coerulea* polyp attachment by ≥ 65%.

The antifouling effects of *C. caroliniana* and *S. californicus* may stem from their chemical compositions, with anti‐adhesion and toxicological effects. Both extracts contained eicosane and 4‐methylphenethylamine, known for their antimicrobial properties [62]. While these compounds likely play a role in antifouling activity, further investigation is required to clarify their specific contributions to fixation inhibition and toxicity. Unique compounds in the *C. caroliniana* extract, such as N‐nonadecanol‐1, cyclohexadecane, and diethanolamine [63], as well as exclusive compounds in the *S. californicus* extract, such as heptadecenal E‐15, 9‐tricosene‐(Z), and piperidine, likely contributed to the antibacterial activity [64], the observed antifouling and toxicological effects. Lysine derivatives, widely found in higher plants, were identified in both extracts and may also have antimicrobial activity [65].

The main chemical groups present, that is, alkanes, aldehydes, alcohols, amines, and amides, play critical roles in plant physiology, influencing plant‐herbivore interactions (pollinator attraction and repellency) and potentially affecting macrofouling organisms' behavior and metabolism [66, 67]. In addition, aldehydes that are part of cellular metabolism and the growth of organisms can be toxic in high concentrations [67]. Therefore, these compounds potentially influence the toxicity and binding of *A. coerulea* through behavioral changes or more complex metabolic changes. For *Aurelia*, little is known about these changes, but in general, for macro‐invertebrates, attachment inhibition may involve neurotransmission disruption, oxidative stress, and inhibition of adhesive production and release [68]. Further studies isolating and testing these compounds individually are necessary to clarify their roles in antifouling efficacy and toxicity. The discrepancy in toxicity levels between these two macrophytes may be attributed to differences in their chemical composition and concentration of specific metabolites [13].

In addition to the antifouling results of the macrophytes *C. caroliniana* and *S. californicus* , through the susceptibility tests with different substances sensitivity, we were also able to observe promising results for the use of *A. coerulea* in toxicological tests. For the chronic toxicity tests with the polyps (tentacle contraction), high values of tentacle contraction were observed in the first observation period with ZS and CC, in addition to the extracts of *C. caroliniana* and *S. californicus* . This response, however, decreased later, with stabilization at 72 and 96 h. At these times, the polyps decreased their maximal contraction responses, especially at lower concentrations. The observed decrease in tentacle contraction may indicate that the polyps were acclimatizing to the conditions of exposure. This phenomenon, known as “functional tolerance” suggests that polyps may be adapting to prolonged exposure, potentially improving their tolerance over time [69]. In other words, exposure to low concentrations can trigger a response that improves tolerance or functional capacity, resulting in an improvement in chronic response over the time of exposure.

In the acute toxicity tests (disintegration), the responses of polyps exposed to the substance's sensitivity (SDS, ZC, and CC) and to the *S. californicus* extract reached stability after 48 h, while for the *C. caroliniana* extract it was only after 96 h. Using the *C. caroliniana* extract, it was possible to verify the importance of performing acute tests within 96 h to evaluate the development of new antifouling agents. This acute disintegration response was also observed by Massaro and Rocha [70]. The authors observed that, for the acute toxicity of adults of the hydrozoan *Hydra viridissima*, disintegration only occurred after 96 h of exposure to potassium dichromate at concentrations of 2.5 and 5 mg L^−1^.

The disparity observed in the LC_50_ ratios between ephyrae and polyps can be attributed to physiological differences between the life cycle stages of this organism. *Aurelia* ephyrae, being at an earlier stage of development, may have different metabolic and detoxification capabilities than polyps, thus influencing their sensitivity to toxic substances. Ephyrae would, therefore, be more sensitive than polyps to certain stressors, such as the microalgae *Ostreopsis* cf. *ovata* [46]. This sensitivity disparity was also observed in trials with saponins and atrazine [33, 71]. In addition, we highlight that in the present study tests with polyps were performed for 96 h, while the tests with ephyrae were performed for 48 h. This discrepancy in the duration of the trials is due to the use of different methodologies, justified by the divergent nature of the two life stages of *A. coerulea* , given the differences in morphology and behavior in the life stages.

When comparing the sensitivity of *A. coerulea* to other organisms, it is important to consider the overlapping confidence intervals for LC_50_/EC_50_ values. This comparison allows the contextualization of *A. coerulea* sensitivity within a broader ecological and toxicological framework. Our study highlights the distinct advantages of using *A. coerulea* polyps and ephyrae in antifouling and toxicological tests. Both *A. coerulea* life stages demonstrated high sensitivity to SDS, ZS, and CC in comparison with other organisms used in toxicological tests (Table 2). The polyps' high sensitivity to SDS was 1.5× to 3.5× greater than that of widely used organisms like the planktonic crustacean *A. salina* (nauplius stage) [53, 55], 5.5× more sensitive than the benthic crustacean *Monokaliapseudes schubarti* [11]. Similarly, polyps were 5.8× more sensitive than the benthic crustacean *Nitokra spinipes* (test with benthic phase; adult) to CC^40^. *Nitokra* species and *M. schubarti* are widely recommended for toxicological testing with water, elutriates, or sediments [72, 73]. These findings suggest that *A. coerulea* is a highly promising benthic model organism for toxicological studies, offering a broader ecological and toxicological perspective.

The *A. coerulea* ephyrae (planktonic phase) also demonstrated good sensitivity when compared to other organisms. It demonstrated 46× greater sensitivity for SDS than *M. schubarti* (adult) [11], 28× higher than *A. salina* (nauplius) [53], 7× higher than *A. amphitrite* (larvae) [74], 6× higher than *A. coerulea* (polyp) (this study). For ZS, the sensitivity of *A. coerulea* ephyra was 3.65× higher than that of *A. salina* [55] and similar to *A. coerulea* polyps (present study). For CC, *A. coerulea* (ephyrae) was 15× more sensitive than polyps (present study), 12× more sensitive than *A. amphitrite* (larvae) [51] and 6× more sensitive than *A. tonsa* [45].

Therefore, we demonstrate that the two stages of the life cycle of *A. coerulea* have different sensitivity, which varies depending on the substance sensitivity used, with greater sensitivity to CC than other tested solutions. This was to be expected, since copper accumulates to a greater degree in scyphozoans than zinc [75], due to its ability to regulate intracellular copper concentrations [76]. While this can happen, metals may not be metabolically available, causing oxidative stress in your tissues, such as increased activity of reactive oxygen species (ROS) due to cellular damage [29]. This may have occurred in the tests performed with copper in the present study, as indicated by the disintegration at low concentrations of this metal. Analyses of ROS activity and lipid peroxidation should, however, be performed to confirm this statement. It is important to note that both the polyps and the ephyrae of *A. coerulea* demonstrate a more pronounced sensitivity than *M. schubarti* and *A. salina* , with a more pronounced sensitivity in the ephyrae.

The results of this study emphasize the potential of the aquatic macrophytes *C. caroliniana* and *S. californicus* as natural antifouling agents. Their extracts effectively inhibited *A. coerulea* polyp attachment without toxicity at practical concentrations (up to 60% for *C. caroliniana* and 15% for *S. californicus* ). We emphasize that the observation (accounting) of attachment polyps was performed only in polyps that were alive, that is, mortality (given by disintegration) did not interfere in this metric analysis, proving that the effect of the extracts was not via toxicity. These compounds can be further extracted, isolated, and incorporated into environmentally friendly antifouling paints [12].

However, some limitations of the present study should be acknowledged. For example, the lack of conditions to prove in which route of action the extracts are acting, since they are not acting in a toxic way, is it an action at the molecular or physiological level? In addition, because we did a preliminary chemical analysis, we were unable to quantify the exact concentration of each compound found in the extracts, which limits knowing which compound is having the most influence on the inhibition of polyp adhesion. Field tests should also be carried out to observe the long‐term effect on the natural environment, where the hydrodynamic and ecological conditions of the environment are complex.

Despite this, the efficiency of *A. coerulea* polyps and ephyrae as test organisms (as shown in this study) offers significant advantages over traditional species, both in terms of assay duration and sensitivity. *A. coerulea* polyps are a better alternative than other macrofouling species because both polyp and ephyrae stages can also be used in toxicological tests. For toxicological tests, *A. coerulea* demonstrated higher sensitivity compared to other widely used model toxicology study organisms, ephyrae sensitivity being more pronounced than that of polyps. By leveraging their unique life cycle, researchers can conduct rapid and comprehensive tests, paving the way for developing less harmful antifouling solutions.

## 5 Conclusion

Research on aquatic macrophyte extracts for biofouling control remains in its early stages, particularly regarding their effects on macrofouling organisms. This study evaluated the antifouling efficacy of aqueous extracts of *C. caroliniana* and *S. californicus* by examining their ability to inhibit the attachment of *A. coerulea* polyps, as well as their toxicity to polyps and ephyrae. It provides critical insights into the antifouling potential of macrophyte extracts, establishing *A. coerulea* as a promising model organism for antifouling and toxicological tests. Extracts from *C. caroliniana* and *S. californicus* inhibited up to 65% of *A. coerulea* polyp attachment while exhibiting no toxicity at practical concentrations (20% for *C. caroliniana* and 5% for *S. californicus* ). Thus, we indicate *C. caroliniana* as the most promising extract among those tested in our study. These results underscore the potential of these extracts as environmentally friendly antifouling agents, paving the way for their use in developing natural antifouling paints.

We also highlight the potential use of *A. coerulea* as a test organism for the evaluation of new antifouling agents, since it can be used for anti‐attachment and toxicological tests (two life cycle stages). Furthermore, *A. coerulea* demonstrated high sensitivity and rapid response times, making it an efficient organism for assessing antifouling and toxicity. Its ease of cultivation and maintenance as well as reproducibility in tests further enhance its utility. For optimal results, we recommend chronic toxicity tests with polyps over 48 h and acute toxicity assessments over 96 h. The *A. coerulea* polyps are suitable for attachment assays, with their maximum response observable within 72 h. This rapid response time could streamline the process of evaluating antifouling agents.

Future research should focus on elucidating the mechanisms behind attachment inhibition through biomolecular studies, field‐testing antifouling efficacy, and evaluating extract stability for incorporation as additives in antifouling paints. Additionally, isolating and characterizing bioactive compounds from the extracts could advance their incorporation into sustainable antifouling solutions. These efforts will contribute significantly to mitigating marine pollution and improving biofouling management in industrial applications.

## Author Contributions

All authors contributed to the study conception and design. The first draft of the manuscript was written by Mikael Luiz Pereira Morales, and all authors commented on previous versions of the manuscript. All authors read and approved the final manuscript.

## Conflicts of Interest

The authors declare no conflicts of interest. Co‐author Ng Haig They received research support from the National Council for Scientific and Technological Development—CNPq (Process 404233/2021‐0). Co‐author Grasiela Lopes Leães Pinho received research support from the Mixed Technical Commission of Salto Grande and Latitud (Uruguayan Technological Laboratory Foundation—LATU).

## Supporting information


**Data S1:** Supporting Information.

## Data Availability

The data that support the findings of this study are available from the corresponding author upon reasonable request.
