# Supplementary material for: Cabomba caroliniana and Schoenoplectus californicus as Antifouling Candidates: Anti‐Attachment and Toxicological Effects in Aurelia coerulea (Cnidaria, Scyphozoa)
Source: Environ Toxicol. 2025 Oct 25;41(6):356–72. doi: 10.1002/tox.24579 (PMC13155321; doi:10.1002/tox.24579)

Appendices (Supplementary material)

**Table S1**: Mean ± SD of attachment and disintegration of *Aurelia coerulea* polyps exposed to Sodium Dodecyl Sulfate (A), Zinc Sulfate (B), Sodium Chloride II (C), *Cabomba caroliniana* extract (D) and *Schoenoplectus californicus* (E).

| **A - Sodium Dodecyl Sulfate (SDS)** | | **Attachment (%)** | | **Disintegration (%)** | |
| --- | --- | --- | --- | --- | --- |
| **Concentration (mg.L^-1^)** | **Observation period (h)** | **Mean** | **SD** | **Mean** | **SD** |
| 0 | 24 | 0.00 | 0.00 | 0.00 | 0.00 |
|  | 48 | 50.00 | 16.67 | 0.00 | 0.00 |
|  | 72 | 100.00 | 0.00 | 0.00 | 0.00 |
|  | 96 | 100.00 | 0.00 | 0.00 | 0.00 |
| 5 | 24 | 0.00 | 0.00 | 0.00 | 0.00 |
|  | 48 | 38.89 | 12.42 | 11.11 | 15.71 |
|  | 72 | 55.56 | 41.57 | 11.11 | 15.71 |
|  | 96 | 88.89 | 15.71 | 11.11 | 15.71 |
| 15 | 24 | 0.00 | 0.00 | 100.00 | 0.00 |
|  | 48 | 0.00 | 0.00 | 100.00 | 0.00 |
|  | 72 | 0.00 | 0.00 | 100.00 | 0.00 |
|  | 96 | 0.00 | 0.00 | 100.00 | 0.00 |
| 45 | 24 | 0.00 | 0.00 | 100.00 | 0.00 |
|  | 48 | 0.00 | 0.00 | 100.00 | 0.00 |
|  | 72 | 0.00 | 0.00 | 100.00 | 0.00 |
|  | 96 | 0.00 | 0.00 | 100.00 | 0.00 |
| 135 | 24 | 0.00 | 0.00 | 100.00 | 0.00 |
|  | 48 | 0.00 | 0.00 | 100.00 | 0.00 |
|  | 72 | 0.00 | 0.00 | 100.00 | 0.00 |
|  | 96 | 0.00 | 0.00 | 100.00 | 0.00 |
| **B - Zinc Sulfate** | | **Attachment (%)** | | **Disintegration (%)** | |
| **Concentration (mg.L^-1^)** | **Observation period (h)** | **Mean** | **SD** | **Mean** | **SD** |
| 0 | 24 | 0.00 | 0.00 | 0.00 | 0.00 |
|  | 48 | 50.00 | 16.67 | 0.00 | 0.00 |
|  | 72 | 100.00 | 0.00 | 0.00 | 0.00 |
|  | 96 | 100.00 | 0.00 | 0.00 | 0.00 |
| 1 | 24 | 0.00 | 0.00 | 0.00 | 0.00 |
|  | 48 | 22.22 | 15.71 | 0.00 | 0.00 |
|  | 72 | 55.56 | 41.57 | 0.00 | 0.00 |
|  | 96 | 88.89 | 15.71 | 0.00 | 0.00 |
| 2 | 24 | 0.00 | 0.00 | 0.00 | 0.00 |
|  | 48 | 22.22 | 15.71 | 0.00 | 0.00 |
|  | 72 | 66.67 | 0.00 | 0.00 | 0.00 |
|  | 96 | 66.67 | 0.00 | 0.00 | 0.00 |
| 3 | 24 | 0.00 | 0.00 | 22.22 | 15.71 |
|  | 48 | 11.11 | 15.71 | 22.22 | 15.71 |
|  | 72 | 44.44 | 15.71 | 33.33 | 0.00 |
|  | 96 | 33.33 | 0.00 | 33.33 | 0.00 |
| 4 | 24 | 0.00 | 0.00 | 55.56 | 15.71 |
|  | 48 | 0.00 | 0.00 | 55.56 | 15.71 |
|  | 72 | 11.11 | 15.71 | 77.78 | 15.71 |
|  | 96 | 11.11 | 15.71 | 77.78 | 15.71 |
| 5 | 24 | 0.00 | 0.00 | 44.44 | 15.71 |
|  | 48 | 0.00 | 0.00 | 88.89 | 15.71 |
|  | 72 | 0.00 | 0.00 | 100.00 | 0.00 |
|  | 96 | 0.00 | 0.00 | 100.00 | 0.00 |
| **C - Cupper Chloride II** | | **Attachment (%)** | | **Disintegration (%)** | |
| **Concentration (mg.L^-1^)** | **Observation period (h)** | **Mean** | **SD** | **Mean** | **SD** |
| 0 | 24 | 0.00 | 0.00 | 0.00 | 0.00 |
|  | 48 | 55.56 | 15.71 | 0.00 | 0.00 |
|  | 72 | 100.00 | 0.00 | 0.00 | 0.00 |
|  | 96 | 100.00 | 0.00 | 0.00 | 0.00 |
| 0.1 | 24 | 0.00 | 0.00 | 0.00 | 0.00 |
|  | 48 | 11.11 | 15.71 | 0.00 | 0.00 |
|  | 72 | 55.56 | 41.57 | 0.00 | 0.00 |
|  | 96 | 100.00 | 0.00 | 0.00 | 0.00 |
| 0.25 | 24 | 0.00 | 0.00 | 0.00 | 0.00 |
|  | 48 | 11.11 | 15.71 | 0.00 | 0.00 |
|  | 72 | 55.56 | 15.71 | 33.33 | 0.00 |
|  | 96 | 44.44 | 15.71 | 33.33 | 0.00 |
| 0.5 | 24 | 0.00 | 0.00 | 0.00 | 0.00 |
|  | 48 | 0.00 | 0.00 | 66.67 | 0.00 |
|  | 72 | 22.22 | 15.71 | 66.67 | 0.00 |
|  | 96 | 22.22 | 15.71 | 66.67 | 0.00 |
| 1 | 24 | 0.00 | 0.00 | 0.00 | 0.00 |
|  | 48 | 0.00 | 0.00 | 100.00 | 0.00 |
|  | 72 | 0.00 | 0.00 | 100.00 | 0.00 |
|  | 96 | 0.00 | 0.00 | 100.00 | 0.00 |
| 2.5 | 24 | 0.00 | 0.00 | 88.89 | 15.71 |
|  | 48 | 0.00 | 0.00 | 100.00 | 0.00 |
|  | 72 | 0.00 | 0.00 | 100.00 | 0.00 |
|  | 96 | 0.00 | 0.00 | 100.00 | 0.00 |
| **D - Cabomba caroliniana** | | **Attachment (%)** | | **Disintegration (%)** | |
| **Concentration (%)** | **Observation period (h)** | **Mean** | **SD** | **Mean** | **SD** |
| 0 | 24 | 0.00 | 0.00 | 0.00 | 0.00 |
|  | 48 | 55.56 | 15.71 | 0.00 | 0.00 |
|  | 72 | 100.00 | 0.00 | 0.00 | 0.00 |
|  | 96 | 100.00 | 0.00 | 0.00 | 0.00 |
| 5 | 24 | 0.00 | 0.00 | 0.00 | 0.00 |
|  | 48 | 11.11 | 15.71 | 0.00 | 0.00 |
|  | 72 | 22.22 | 15.71 | 0.00 | 0.00 |
|  | 96 | 22.22 | 15.71 | 0.00 | 0.00 |
| 10 | 24 | 0.00 | 0.00 | 0.00 | 0.00 |
|  | 48 | 11.11 | 15.71 | 0.00 | 0.00 |
|  | 72 | 11.11 | 15.71 | 0.00 | 0.00 |
|  | 96 | 11.11 | 15.71 | 0.00 | 0.00 |
| 20 | 24 | 0.00 | 0.00 | 0.00 | 0.00 |
|  | 48 | 22.22 | 15.71 | 0.00 | 0.00 |
|  | 72 | 33.33 | 0.00 | 0.00 | 0.00 |
|  | 96 | 33.33 | 0.00 | 0.00 | 0.00 |
| 40 | 24 | 0.00 | 0.00 | 0.00 | 0.00 |
|  | 48 | 11.11 | 15.71 | 0.00 | 0.00 |
|  | 72 | 11.11 | 15.71 | 0.00 | 0.00 |
|  | 96 | 11.11 | 15.71 | 22.22 | 31.42 |
| 80 | 24 | 0.00 | 0.00 | 0.00 | 0.00 |
|  | 48 | 0.00 | 0.00 | 33.33 | 0.00 |
|  | 72 | 0.00 | 0.00 | 44.44 | 15.71 |
|  | 96 | 0.00 | 0.00 | 44.44 | 15.71 |
| **E - Schoenoplectus californicus** | | **Attachment (%)** | | **Disintegration (%)** | |
| **Concentration (%)** | **Observation period (h)** | **Mean** | **SD** | **Mean** | **SD** |
| 0 | 24 | 0.00 | 0.00 | 0.00 | 0.00 |
|  | 48 | 100.00 | 0.00 | 0.00 | 0.00 |
|  | 72 | 100.00 | 0.00 | 0.00 | 0.00 |
|  | 96 | 100.00 | 0.00 | 0.00 | 0.00 |
| 5 | 24 | 0.00 | 0.00 | 0.00 | 0.00 |
|  | 48 | 22.22 | 15.71 | 0.00 | 0.00 |
|  | 72 | 66.67 | 0.00 | 0.00 | 0.00 |
|  | 96 | 66.67 | 0.00 | 0.00 | 0.00 |
| 10 | 24 | 0.00 | 0.00 | 0.00 | 0.00 |
|  | 48 | 22.22 | 15.71 | 11.11 | 15.71 |
|  | 72 | 33.33 | 27.22 | 22.22 | 15.71 |
|  | 96 | 33.33 | 27.22 | 22.22 | 15.71 |
| 20 | 24 | 0.00 | 0.00 | 0.00 | 0.00 |
|  | 48 | 22.22 | 15.71 | 22.22 | 15.71 |
|  | 72 | 22.22 | 31.43 | 44.44 | 15.71 |
|  | 96 | 22.22 | 31.43 | 44.44 | 15.71 |
| 40 | 24 | 0.00 | 0.00 | 0.00 | 0.00 |
|  | 48 | 0.00 | 0.00 | 77.77 | 15.72 |
|  | 72 | 0.00 | 0.00 | 88.89 | 15.72 |
|  | 96 | 0.00 | 0.00 | 88.89 | 15.72 |
| 80 | 24 | 0.00 | 0.00 | 0.00 | 0.00 |
|  | 48 | 0.00 | 0.00 | 100.00 | 0.00 |
|  | 72 | 0.00 | 0.00 | 100.00 | 0.00 |
|  | 96 | 0.00 | 0.00 | 100.00 | 0.00 |

**Table S2**: GC-MS analysis of aqueous extract of aquatic macrophytes (*Cabomba caroliniana* and *Schoenoplectus californicus*).

| **GC-MS Analysis** | | | | | | |
| --- | --- | --- | --- | --- | --- | --- |
|  |  | ***C. caroliniana* extract** | | ***S. californicus* extract** | |  |
| **Compound** | **Molecular Formula** | **R.Time** | **Area %** | **R.Time** | **Area %** | **Chemical group** |
| 1-Dodecen-3-ol | C_12_H_24_O | - | - | 21.310 | 0.34 | Alcohol |
| 1-Hexen-4-ol. 4-cyclohexyl-3-methyl | C_13_H_24_O | - | - | 11.310 | 0.88 | Alcohol |
| 1H-Tetrazole-1-ethano | C_3_H_6_N_4_O | 13.435 | 3.16 | - | - | Alcohol |
| 1-Pentadecene | C_15_H_30_ | - | - | 11.249 | 4.32 | Alkene |
| 1-Tetradecene | C_14_H_28_ | 11.266 | 7.08 | - | - | Alkene |
| 5.5-Diethylpentadecane | C_19_H_40_ | 14.746 | 3.48 | 14.050 | 0.39 | Alkane |
| 5-Hydroxy-6-methyl-12.13-dioxa-tricyclo | C_14_H_22_O_5_ | 18.495 | 2.15 | - | - | Alcohol |
| 5-Methyldodecane | C_13_H_28_ | - | - | 7.103 | 1.47 | Alkane |
| 9-Tricosene | C_23_H_46_ | - | - | 15.321 | 13.94 | Alkene |
| alpha.-D-Xylofuranose. cyclic 1.2:3.5-bis(methylboronate) | C_7_H_12_B_2_O_5_ | 21.395 | 1.97 | - | - | Boronated carbohydrate |
| Cyclohexadecane | C_16_H_32_ | 13.334 | 19.42 | - | - | Ciclic Alkane |
| Decane. 5.6-dimethyl- | C_12_H_26_ | - | - | 15.465 | 0.54 | Alkane |
| Dodecane. 2.6.11-trimethyl | C_15_H_32_ | 9.860 | 3.32 | 9.846 | 5.41 | Alkane |
|  |  | - | - | 10.405 | 2.11 |  |
|  |  | - | - | 11.945 | 1.52 |  |
| E-15-Heptadecenal | C_17_H_32_O | - | - | 13.325 | 16.08 | Aldehyde |
| Eicosane | C_20_H_42_ | 12.230 | 5.08 | 12.220 | 8.37 | Alkane |
|  |  | 14.317 | 5.07 | 12.319 | 1.50 |  |
|  |  | 17.536 | 2.63 | 12.694 | 8.01 |  |
|  |  | - | - | 14.313 | 6.96 |  |
|  |  | - | - | 14.744 | 3.08 |  |
|  |  | - | - | 16.819 | 3.11 |  |
|  |  | - | - | 17.528 | 1.87 |  |
| Heptane | C_7_H_16_ | - | - | 4.302 | 0.59 | Alkane |
| Hexadecane. 1-iodo- | C_16_H_33_Eu | - | - | 12.805 | 2.10 | Alkane |
| Hexadecane. 2.6.11.15-tetramethyl- | C_20_H_42_ | 12.699 | 5.18 | - | - | Alkane |
| Methoxyacetic acid. 4-hexadecyl ester | C_19_H_38_O_3_ | - | - | 21.383 | 2.73 | Ester |
| Methyl 13.16-docosadienoate | C_23_H4_2_O_2_ | 18.065 | 3.58 | - | - | Methyl ester |
| N-Methyl-10-hydroxydecahydroquinoline | C_10_H_19_NO | 16.876 | 1.87 | - | - | Quinoline |
| n-Nonadecanol-1 | C_19_H_40_O | 15.331 | 16.67 | 18.366 | 3.42 | Alcohol |
|  |  | 18.376 | 7.72 | - | - |  |
| Nonane. 5-methyl-5-propyl | C_13_H_28_ | - | - | 10.515 | 0.79 | Alkane |
| Octadecane. 5-methyl | C_18_H_38_ | 16.830 | 2.33 | 12.900 | 0.49 | Alkane |
| Phenol. 2.4-bis(1.1-dimethylethyl)- | C_14_H_22_O | 12.597 | 5.49 | 12.586 | 4.51 | Fenol |
| Piperidine. 1-(1.2.3.4-tetrahydro-2-naphthaleny | C_23_H_26_FNO | 18.284 | 1.77 | - | - | Piperidine |
| Toluene | C_7_H_8_ | - | - | 3.886 | 1.48 | Aromatic compounds |
| Tridecanol. 2-ethyl-2-methyl- | C_16_H_34_O | - | - | 14.976 | 1.07 | Alcohol |
| Undecane. 2-methyl- | C_12_H_26_ | - | - | 13.435 | 2.01 | Alkane |
| Z-5-Methyl-6-heneicosen-11-one | C_22_H_42_O | 17.280 | 2.00 | - | - | Ketone |

**Table S3:** LC-MS analysis of aqueous extract of aquatic macrophytes (*Cabomba caroliniana* and *Schoenoplectus californicus*).

|  |  | ***C. caroliniana* extract** | | | ***S. californicus* extract** | | |  |
| --- | --- | --- | --- | --- | --- | --- | --- | --- |
| **Compound** | **Molecular Formula** | **R.Time** | **Area %** | **Base Peak m/z** | **R.Time** | **Area %** | **Base Peak m/z** | **Chemical group** |
| 2-Trifluoromethyl-benzenesulfonamide | C_7_H_6_F_3_NO_2_S | 5.097 | 0.56 | 224.80 | 5.142 | 0.28 | 224.80 | Aromatic compound |
| 4-Methylphenethylamine | C_9_H_13_N | 4.694 | 32.75 | 135.55 | 4.783 | 41.84 | 135.55 | Aromatic compound |
|  |  | 5.109 | 45.10 |  | 5.133 | 31.99 |  |  |
| Acridone | C_13_H_9_NO | 4.492 | 0.34 | 194.90 | 4.473 | 0.37 | 194.90 | Aromatic compound |
|  |  | 4.768 | 0.27 |  |  |  |  |  |
| Benzamidine hydrochloride | C_7_H_9_ClN_2_ | 0.494 | 0.13 | 121.10 | - | - | - | Aromatic compound |
| D-cysteine | C_3_H_7_NO_2_S | 4.325 | 0.44 | 121.15 | - | - | - | Amino acid |
| Diethanolamine | C_4_H_11_NO_2_ | 5.342 | 3.78 | 105.20 | - | - | - | Amines |
|  |  | 5.501 | 15.51 |  |  |  |  |  |
|  |  | 35.580 | 0.06 |  |  |  |  |  |
| Lysine Hydrochloride | C_6_H_14_N_2_O_2_ClH | 33.434 | 0.10 | 146.20 | 6.367 | 0.03 | 146.20 | Amino acid |
|  |  | 35.168 | 0.11 |  | 22.855 | 0.06 |  |  |
|  |  | 37.267 | 0.10 |  |  |  |  |  |
|  |  | 37.512 | 0.12 |  |  |  |  |  |
|  |  | 37.967 | 0.35 |  |  |  |  |  |
|  |  | 38.343 | 0.09 |  |  |  |  |  |
|  |  | 39.023 | 0.17 |  |  |  |  |  |
| Benzaldehyde | C_7_H_6_O | - | - | - | 5.425 | 3.52 | 106.25 | Aromatic compound |
| Isonicotinic acid | C_6_H_5_NO_2_ | - | - | - | 5.367 | 1.76 | 123.15 | Carboxylic acid |
| L-Lysine monohydrochloride | C_6_H_15_ClN_2_O_2_ | - | - | - | 5.608 | 13.42 | 146.15 | Amino acid |
|  |  |  |  |  | 28.118 | 0.03 |  |  |
|  |  |  |  |  | 18.300 | 0.05 |  |  |
|  |  |  |  |  | 28.571 | 0.04 |  |  |
|  |  |  |  |  | 29.213 | 0.09 |  |  |
|  |  |  |  |  | 32.372 | 0.06 |  |  |
|  |  |  |  |  | 33.300 | 0.04 |  |  |
|  |  |  |  |  | 33.706 | 0.06 |  |  |
| Piperidinic acid | C_4_H_9_NO_2_ | - | - | - | 4.373 | 6.36 | 103.30 | Carboxylic acid |

**Table S4:** FTIR analysis of aqueous extract of aquatic macrophytes (*Cabomba caroliniana* and *Schoenoplectus californicus*).

|  |  | ***C. caroliniana* extract** | ***S. californicus* extract** |
| --- | --- | --- | --- |
| **Phytocompounds identified** | **Functional groups** | **Wave number (cm^-1^)** | **Wave number (cm^-1^)** |
| Primary Amine | N-H stretch | 3334.58 | - |
| Aldehyde | C-H | 2977.48 | - |
| Amide | C=O stretch/ Aliphatic C=C stretching | 1636.38 | 1633.9 |
| Fluoro Compound | C-F stretch | 1100.11 | - |
| Alcohol-Hydrogen bonded | O-H stretch | - | 3353.08 |
| Alkyne | C≡C stretch | - | 2105.96 |
| Alcohol | O-H Bend / N-H deformation | - | 1418.2 |
| Aliphatic Ether | C-F Stretch/ C-O | - | 1106.67 |
| Halo Compound | C-Br | - | 603.8 |

**Figure S1:** GC-MS analysis. peaks of aqueous extract of aquatic macrophytes. A - *Cabomba caroliniana*; B - *Schoenoplectus californicus*.


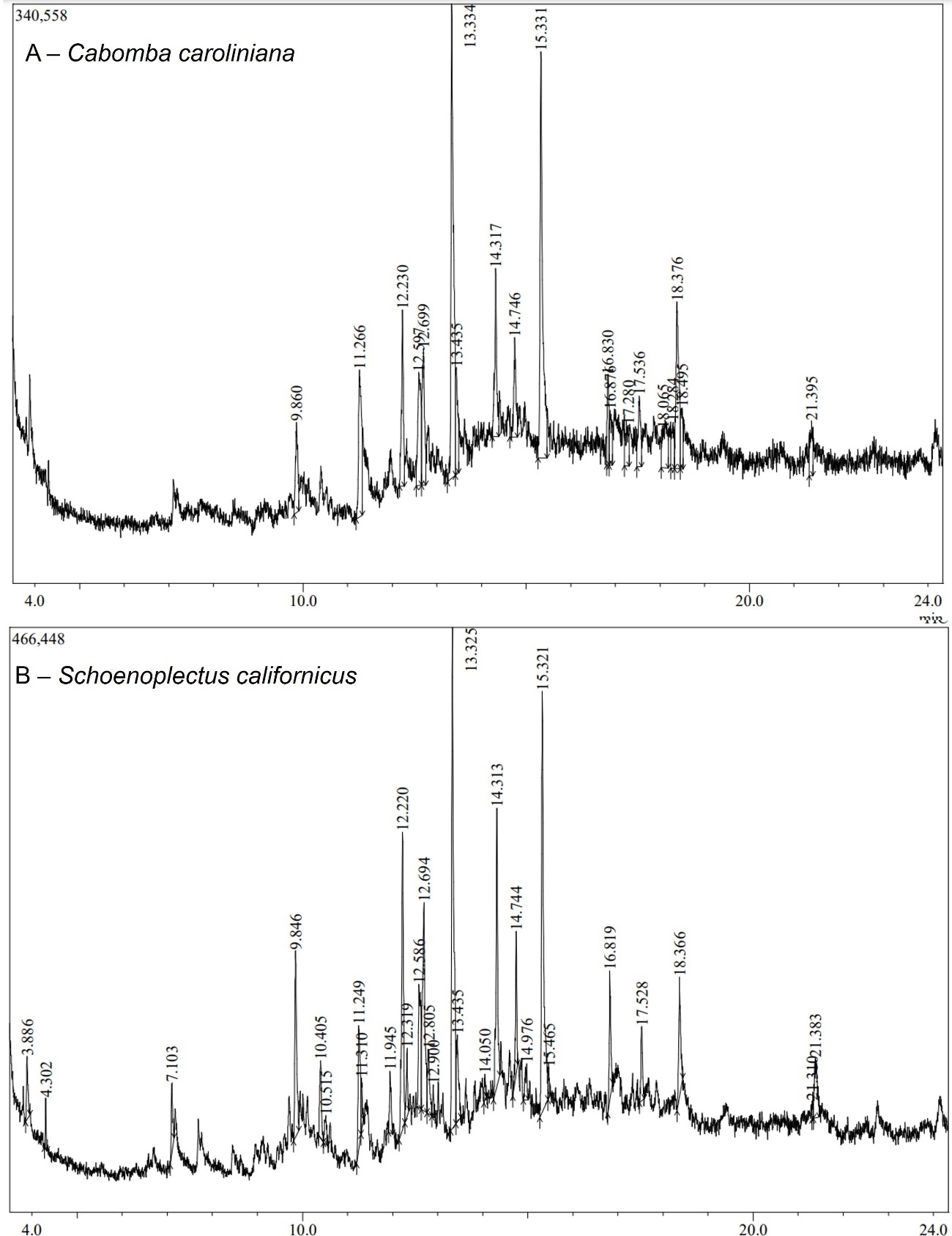


**Figure S2:** LC-MS analysis. peaks of aqueous extract of aquatic macrophytes. A - *Cabomba caroliniana*; B - *Schoenoplectus californicus*.


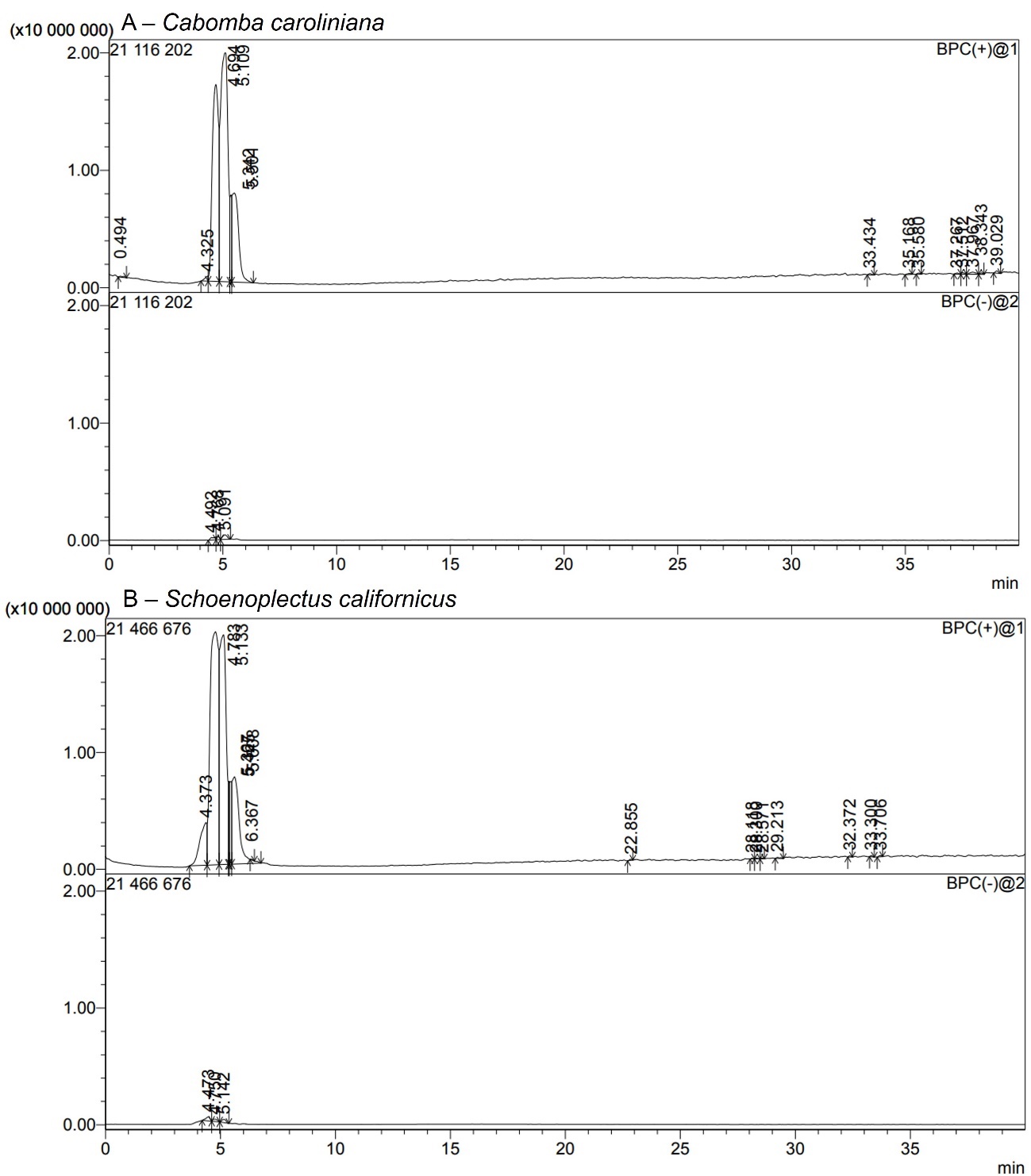

Supplement: Supplementary file 1 — Data S1: Supporting Information. [file TOX-41-356-s001.docx]
